# Efficacy of disease management program used among patients with chronic heart failure: protocol for a systematic review and network meta-analysis

**DOI:** 10.1186/s13643-023-02183-8

**Published:** 2023-02-28

**Authors:** Yongjie Duan, Zongren Li, Qin Zhong, Chongyou Rao, Yun Hua, Rilige Wu, Jing Dong, Da Li, Wenjun Wang, Kunlun He

**Affiliations:** 1 The Medical School of Chinese PLA, Chinese PLA General Hospital, Beijing, 100853 China; 2 Center for Artificial Intelligence in Medicine, Chinese PLA General Hospital, Beijing, 100853 China; 3 Medical Big Data Research Center, Chinese PLA General Hospital, Beijing, 100853 China; 4 Bio-Engineering Research Center, Chinese PLA General Hospital, Beijing, 100039 China; 5 Medical Engineering Laboratory, Chinese PLA General Hospital, Beijing, 100048 China

**Keywords:** Heart failure, Management program, Meta-review

## Abstract

**Background:**

A large number of studies have provided a variety of heart failure management program (HF-MP) intervention modes. It is generally believed that HF-MP is effective, but the question of which type of program works best, what level of support is needed for an intervention to be effective, and whether different subgroups of patients are best served by different types of programs is still confusing.

**Methods:**

This study will search for published and unpublished randomized clinical trials in English examining HF-MP interventions in comparison with usual care. MEDLINE, Medlin In-Process and Non-Indexed, CENTRAL, CINAHL, EMBASE, and PsycINFO will be the databases. We will calibrate our eligibility criteria among the team. Each literature will be screened by at least two reviewers. Conflicts will be resolved through team discussion. A similar process will be used for data abstraction and quality appraisal. The results will be synthesized descriptively, and a network meta-analysis will be conducted if the studies are deemed methodologically, clinically, and statistically acceptable (e.g., *I*^2^ < 50%). Moreover, potential moderators of efficacy will be analyzed using a meta-regression.

**Discussion:**

This study will reduce the clinical heterogeneity and statistical heterogeneity of review and meta-analysis through a more scientific classification method to determine the most effective HF-MP in different subgroups of heart failure patients with different human resource investments and different intervention methods, providing high-quality evidence and guidance for clinical practice.

**Systematic review registration:**

PROSPERO CRD42021258521

**Supplementary Information:**

The online version contains supplementary material available at 10.1186/s13643-023-02183-8.

## Background

Heart failure (HF) is one of the most common chronic diseases. An estimated 64.3 million people are living with HF worldwide [1]. Despite the continuous development of various treatments, the rates of morbidity, mortality, and healthcare expenditure associated with HF remain high. The latest European Society of Cardiology (ESC) guidelines recommends that patients with HF enroll in a multidisciplinary team heart failure management program (HF-MP) and self-management strategies to reduce the risk of HF hospitalization and mortality [2]. However, it has also been reported that almost 40% of early readmissions in patients with HF may be related to suboptimal disease management programs [3, 4], which shows that different disease management programs can vary considerably in their effects on outcomes.

Since the concept of “disease management programs” was established in the 1990s, a variety of models have emerged in the field of HF-MP, which have differences in terms of intervention content, healthcare professional involvement, and delivery methods. Although several large randomized clinical trial (RCT) studies recently published show contradictory results, HF-MPs are generally considered useful. Some systematic reviews have been published before to try to synthesize results, evaluating case-management, multidisciplinary, pharmacy-led, and other interventions; none have ranked all of the available intervention. Due to the limitation of sample size (most studies are less than 5000 people) [5–11], the method limitations of pair-wise itself, and the high heterogeneity of comprehensive intervention categories [6, 8–16], these systematic reviews cannot integrate all intervention models to obtain high quality, and quantitative conclusions. In addition, no studies have assessed the huge investment in health resources associated with complex case management programs. So far, the issue of “which type of program works best, what level of support is needed for an intervention to be effective, and whether different subgroups of patients are best served by different types of programs” has not been discussed clearly. Although only one network meta-analysis (NMA) has been published that compared interventions into seven groups and concluded that “nurse home visits” and “disease management programs” are effective in reducing all-cause mortality, it has not yet concluded which model is more effective. The authors emphasized the need to synthesize larger evidence and minimize heterogeneity in intervention categories [17].

Policymaker and clinicians continue to make decisions without strong and specific evidence. Several recent large RCTs may provide us with updated evidence. High-quality supporting evidence needs to overcome the clinical and statistical heterogeneity that has mired previous systematic reviews by appropriately distinguishing treatment types, and also requires larger sample sizes and more rigorous methods to detect intervention component details. Therefore, intervention types will be categorized on the basis of content, healthcare professional involvement, frequencies of encounters, and delivery methods to investigate the effectiveness of different HF-MP models through a NMA including exhaustive searches of published and publicly available unpublished information. Furthermore, we will use the taxonomy developed by the American Heart Association (AHA) [18] to provide a comprehensive assessment of the content, components, providers, and standardized procedures that likely influence treatment effectiveness. We will assess in detail how patient-level data interacted with the model of intervention and the site of implementation. The purpose of this study is to determine the effect of different HF-MP in different subgroups of HF patients and inform policymakers about which models are appropriate for widespread implementation.

## Methods

**Fig. 1:**
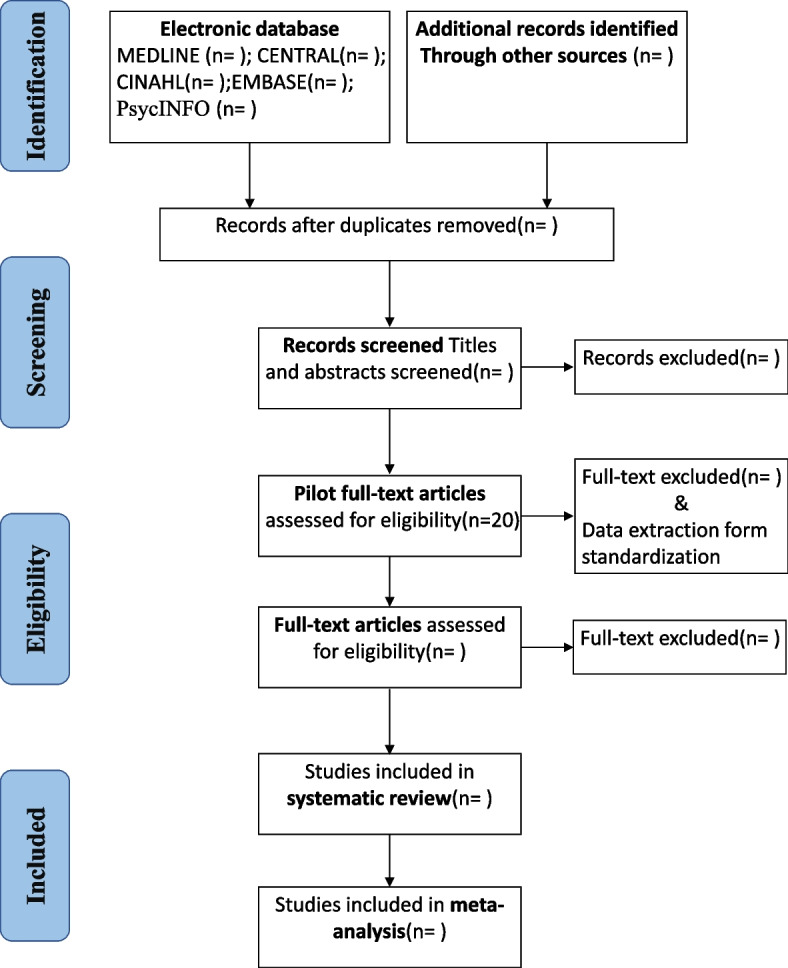
The PRISMA flow diagram of the study selection process

The team compiled this protocol under the guidance of the Preferred Reporting Items for Systematic Reviews and Meta-analyses Protocols (PRISMA-P) [19] (Fig. 1) and the Cochrane Handbook for Systematic Reviews of Interventions (Version 6) [20]. This protocol was registered in the PROSPERO database (CRD42021258521; available at https://www.crd.york.ac.uk/prospero/#myprospero).

### Eligibility criteria

The eligibility criteria are under the Population-Intervention-Comparison-Outcomes-Study type (PICOS):

#### Population

Studies enrolling adult patients (aged 18 years and older) hospitalized with a primary diagnosis of HF who were recruited either before or within 7 days after discharge will be included. People whose primary diagnosis was not HF will be excluded. Since HF patients with psychiatric disorders, such as depression, do not cooperate well with HF-MP, RCTs whose study population was patients with HF and psychiatric disorders will be excluded. To reduce heterogeneity, RCTs which were primarily designed to deal with the problems of caregivers (e.g., medical staff, social workers, pharmacists, family member) rather than patients will also be excluded.

#### Intervention

Disease management interventions directed specifically at HF patients will be included. Intervention content can involve one or more components, such as education, follow-up, remote monitoring, and drug adjustment. The specific definition of components of HF-MP in this study is shown in the Supplementary Materials. Although most implanted devices have remote monitoring capabilities, RCTs involving implanted devices will be excluded because we believe that implantable devices should be classified as another major type of treatment for HF. They had no direct effect on the mastery of patient-related knowledge or active mobilization. RCTs that focus on evaluating the efficacy of different drugs or medical apparatus will be excluded. Although exercise rehabilitation is important, RCTs which only involved an exercise rehabilitation program will be excluded because of the large differences in exercise type, volume, and effect evaluation indicators. RCTs in which interventions were administered only during hospitalization are not fully consistent with the concept of HF-MP and, therefore, will be excluded.

#### Comparison

Eligible comparators will include usual care.

#### Outcomes

Studies will have to examine the primary or secondary outcomes of interest identified below. The primary outcomes include all-cause mortality and HF-related readmission. Secondary outcomes include all-cause readmission, HF-related mortality, the number of emergency department visits, and quality of life (QOL). QOL will be measured using the Kansas City Cardiomyopathy Questionnaire (KCCQ) overall summary score [21], Minnesota Living with Heart Failure Questionnaire (MLHF) global score [22], 12-item Short Form Health Survey(SF-12) [23], and 36-Item Short Form Health Survey questionnaire(SF-36) [24]. Studies with none of the outcomes mentioned above will be excluded.

#### Study type

This project will identify all types of RCTs (such as crossover, cluster, and patient-randomized clinical trials) published from January 2000 to August 2021 where a HF-MP intervention was tested. Non-English studies will be excluded unless they have an English-language version of the abstract containing sufficient data for effect-size calculations. All other study designs will be excluded.

### Information sources and search strategy

We will begin by searching electronic databases in MEDLINE (the US National Library of Medicine’s database), MEDLINE In-Process and Other Non-Indexed Citations, the Cochrane Central Register of Controlled Trials (CENTRAL), Cumulative Index to Nursing and Allied Health (CINAHL), Embase, and PsycINFO (the Behavioral Science and Mental Health Database) from January 2000 to August 2021.

The year 2000 cutoff was chosen since this marked the emergence of remote monitoring, medical therapies such as CRT, and more widespread use of beta-blockers. Meanwhile, the KCCQ, a highly influential and widely used measurement scale for QOL, was published. A combination of MeSH and text terms including HF, management program, and telemonitoring will be used. The draft literature search for the main database (MEDLINE) will be peer-reviewed by another experienced librarian using the Peer Review of Electronic Search Strategies (PRESS) checklist [25]. After this exercise, the literature search will be modified as required. The specific search strategy for MEDLINE is shown in the Supplementary Materials.

-Unpublished studies will also be searched, as the primary difference between published and unpublished studies is not their methodological quality but rather the significance of their results. These searches will include research registries (e.g., clinicaltrials.gov) and conference abstracts to identify unpublished studies.

### Data extraction and management

The documents searched in the database will be managed by using EndNote X9 and Zotero 6.0.18. The statistician (ZRL) will randomly number the papers and assign them to each researcher. The data recording form will be developed by all team members after discussions based on a modified version of the Cochrane Public Health Group’s data extraction template. Each reviewer will maintain and edit their record table locally, and all data will be synchronized on the central server daily. Each article will be reviewed by two independent reviewers. Any conflicts will be resolved through discussion with a third reviewer. All papers reviewed by all eight reviewers intersect each other, that is, each reviewer will screen the articles from all other reviewers. All data management procedures will be performed using Python 3.8.

### Study selection process

The article will not be included in the final analysis until it passes through abstract screening and detailed review.

In the abstract screening phase, research team members will independently screen the initial search results and compare them against the inclusion criteria. At this stage, titles and abstracts will be examined to determine whether the study includes HF-MP. Eligible studies will be marked for full-text retrieval. Correspondingly, non-eligible studies will be excluded, and the reasons for ineligibility will be noted. Similarly, any study that appears to lack sufficient data to calculate an effect size will not be excluded until all searches for other publications on the same study are completed and the study authors are contacted to request additional data. Studies will be excluded at this stage only if there is explicit agreement among all reviewers.

In the detailed review phase, we will first select 20 articles for a preliminary reading in batches and discuss them to continuously improve our spreadsheet until all reviewers reach an agreement. All data entries will be compared between reviewers, and disagreements will be resolved by discussion with a third reviewer. In the case of incompleteness or doubts about any data, the principal investigators will be contacted.

### Data collection process

Pilot extraction was performed on a random sample of 20 studies (concurrent with the detailed review phase mentioned above). Data abstraction was modified as required. Formal extraction will not start until a sufficient agreement is noted (i.e., > 95% agreement). Using a standardized data abstraction form, data will be independently abstracted by two reviewers, who will then make data comparisons until 100% agreement is reached for every item.

### Data items and outcomes

After the selection process, we will abstract data from the included RCTs on study characteristics (e.g., year of conduct, location, sample size, study setting, intervention, and comparator) and participant characteristics (e.g., sex, mean age and standard deviation, HF classification, comorbidities). Different from the previous meta-analysis, the intervention duration and frequency data of different RCTs will also be abstracted. The intervention details for the control group in different RCTs will also be abstracted and evaluated, rather than just summarized as “usual care.” As mentioned above, the following clinical outcomes will be extracted: morality, readmission, healthcare use, and QOL. Each study does not necessarily contain all clinical outcomes, so the final list of outcomes to be analyzed will be determined by the extent of reported and usable data. Meanwhile, different outcomes will be abstracted from each time point reported across RCTs to examine the effects of the interventions. Data from multiple publications of the same research will be integrated, and extraction will only be conducted on RCTs reporting the primary outcome of interest and/or the longest duration of follow-up. Sufficient data will be collected to allow careful judgment of the homogeneity and similarity of assumptions for the meta-analysis, as described in the synthesis section below.

### Risk of bias assessment

The Cochrane Collaboration’s Risk for Bias tool [26] will be used to address the problem of study quality, which permits grading each domain of potential bias as “low risk,” “high risk,” or “unclear risk.” The full text of each eligible study will then be evaluated. The Cochrane Handbook section 16.3.2 and the revised Risk of Bias tool (version 2.0) will be used to assess cluster RCTs [20, 27]. Since blinding of participants is not feasible in lifestyle intervention research [28], the risk of bias will be calculated according to 5 domains: randomization, allocation concealment, blinding of outcome assessment, incomplete outcome data, and selective outcome reporting. The risk of bias graph and the risk of bias summary will be generated by RevMan V.5.3.

In addition, the Grading of Recommendations Assessment, Development and Evaluation (GRADE) [29] guidance will be applied to assess the certainty of evidence (confidence in evidence, quality of evidence) contributing to the NMA.

### Synthesis of included studies

#### HF-MP types and network treatment nodes

We will first classify all the arms of all studies according to the definition of HF-MP types and the intervention details collected from all studies. We plan to divide the HF-MP intervention into five categories based on the intensities of content, the frequencies of encounters, and the primary care provider, as follows: high-intensity hybrid disease management (high-HDM), low-intensity hybrid disease management (low-HDM), high-intensity self-care support (high-SCS), low-intensity self-care support (low-SCS), and self-monitoring (SM). Details of the definitions of these HF-MP programs are provided in Table 1.

**Table 1:** Interventions models (five HF-MP types) and comparator groups, with descriptions

Intervention model						
**Nickname**	**Type**	**Professional involvement degree**	**Frequency**	**Encounter type**	**Delivery personnel**	**Main content**
High-HDM	Hybrid disease management	High	≥ 1/month or ≥ 6 h over the study period	Face-to-face and/or telephone	Healthcare professional team led by HF cardiologist and/or HF nurse, and pharmacy	Review patient status and data; medication reconciliation; facilitating access to care
High-SCS	Self-care support	Moderate	≥ 1/month or ≥ 6 h over the study period	Face-to-face and/or telephone	Healthcare team coordinated by nurse, physician, psychologist, health educator, or trained volunteer	Review patient status, provide Education program and self-care support
Low-HDM	Hybrid disease management	Moderate	< 1/month	Face-to-face and/or telephone	Healthcare professional team led by HF cardiologist and/or HF nurse, and pharmacy	Review patient status and data; medication reconciliation; facilitating access to care
Low-SCS	Self-care support	Low	< 1/month	Face-to-face and/or telephone	Healthcare team coordinated by nurse, physician, psychologist, health educator, or trained volunteer	Review patient status, provide Education program and self-care support
SM	Self-monitoring		No scheduled encounters; real time self-monitoring	Telemonitoring	Self-management	No scheduled interactions with healthcare professionals
**Comparator group**						
EUC	Consisted of the patients being given care guided by local practice that may or may not have included scheduled follow-up visits but did not include any structured educational programs					
UC	The necessary support was provided at the discretion of clinicians that may or may not have included scheduled follow-up visits but did not include any structured educational programs					

In brief, high-intensity programs were defined as those in which encounters with the patients occurred more than once per month or more than 6 h over the study period. Low intensity was defined as patient encounters occurring less than once per month during the intervention period. Hybrid disease management programs were led by health care professionals (including HF specialists, HF nurses, or pharmacists), including review of patients’ clinic status, medication reconciliation, patient education, and facilitated access to care and/or social and psychological support. Self-care support was primarily delivered by an allied healthcare professional team that included physicians, nurses or psychologists, health educators, and/or trained volunteers who provide support and educational programs to enhance patients’ self-care skills and address potential barriers to self-care. Self-monitoring programs were based on telemonitoring.

To remove the bias caused by the different strength of “usual care” groups in different studies, comparator groups will be characterized as either “usual care” (UC) or “enhanced usual care” (EUC). “Usual care” was defined as care provided at the discretion of clinicians and may or may not have included scheduled follow-up visits but did not include any structured educational programs. “Enhanced usual care” was defined as patients being given a formal plan with scheduled follow-up visits and/or being given structured educational material only before they were discharged from the hospital (i.e., no structured educational program following discharge). We will also present the results of not performing this operation as a sensitivity analysis.

#### Bayesian hierarchical NMA

After this, the differences between these interventions will be compared using the Bayesian hierarchical NMA. Fixed- or random-effects models will be selected based on the smaller value of the deviance information criterion (DIC). Non-informative vague priors were used for all parameters.

For the multi-arm study, we will account for the correlations induced by multi-arm studies by employing multivariate distributions. Studies whose groups are the same type of intervention will be removed. If there are identical studies in the multi-arm study, we will divide these studies into several groups of pairwise studies and compare them with each other.

#### Measures of treatment effect

The available data on the statistical outcome of each study will be used. For the two-category variables, in order to eliminate the impact of different follow-up lengths of different studies, we will use hazard ratio (HR) as a measure. For the study of unreported data, the average survival and the number of events will be used. We will count participants who had not been lost follow-up in the time-point their outcome information was reported to calculate the effect size. The effect size for dichotomous outcomes will be the HR and its 95% credible interval, in order to eliminate the bias caused by different follow-up lengths in different studies.

If the original study investigators reported event counts only, differences in follow-up duration between studies were incorporated using the trial patient-year follow-up to estimate HRs using the Poisson likelihood and log link. To verify the reliability, the odds ratio (OR) value in different time periods will be also calculated as supplementary analysis and sensitivity analysis. If there is a zero cell, we will use a continuity correction to add an increment of 0.5 in all cells that are zero.

The effect estimates will choose the standardized mean difference (SMD) for continuous outcomes measured on different scales. Since the change trend of MLHF is opposite to that of KCCQ, SF-12, and SF-36 (the higher the former value corresponding to the worse QOL), a negative number of SMD as the effect size for the studies using the MLHF scale will be taken.

#### Assessment of transitivity and inconsistency

The main clinical characteristic of all nodes and edges in the network will be reported to assess the clinical heterogeneity of interventions and similarity of comparisons, including age, length of intervention, setting (home-based or community-based), mode of delivery (remote or face-to-face or mixed), the provider (nurses, physicians or multidisciplinary), and the severity of HF at baseline, and the results will be present in boxplots.

Inconsistency of direct and indirect results will be evaluated by node-splitting analysis for all comparison loops and indirect results were derived from direct and network results by the back-calculation method. Global and local statistical heterogeneity was assessed with generalized Cochran’s Q. If there are inconsistencies in our network, we will check the data and try to explain it by meta-regression or subgroup analysis and conduct sensitivity analysis to exclude studies.

#### Results of NMA

We will use the surface under the cumulative ranking curve (SUCRA) to rank the HF-MP types for each outcome, and use the league table to present the results of the network meta-analysis comparing the effects of all models. Forest plots with *I*^2^ (with test-based 95% confidence intervals) and heterogeneity variance (*τ*) display statistical heterogeneity.

#### Meta-regression and subgroup NMA

Furthermore, we will do three models of meta-regression on both outcomes involving all studies to assess the effects of important covariates (an additive main effect model that assumes that the effect of each component adds, a two-way interaction model, and a full interaction model for interventions). Covariates in meta-regression-based NMA that we may explore include age, length of intervention, the severity of HF at baseline (average NYHA scores), the delivery personnel (multidisciplinary or not), access to professional care (be able to call doctors or not), and enrollment of caregivers. Subgroup NMA will be conducted according to the meta-regression results.

#### Publication bias and confidence in evidence

Publication bias in the NMA will be investigated by visual inspection of comparison-adjusted funnel plots for asymmetry. And a web application of the GRADE approach will be performed to evaluate the confidence in cumulative evidence, which includes the risk of bias criteria, indirectness, imprecision, inconsistency, and publication bias.

#### Sensitivity analysis

A series of sensitivity analysis will be conducted to examine the effects, which refer to excluding trials with high or unknown risk of bias, excluding quasi-randomized or cluster randomized trials, excluding trials with a follow-up of less than 3 months or more than 2 years, excluding unpublished trials, using random effect models, combining the UC and EUC group, and using RR as effect size in different time periods.

#### Statistical details

The NMA will be conducted using Gemtc, an R package to conduct Bayesian NMA used to build complex statistical models using Markov chain Monte Carlo simulation.

## Discussion

Characterized by the low quality of life, high medical costs, and premature death, HF has become a modern epidemic with a huge economic and human burden on the community [30, 31]. Although guidelines provide key components for self-management for HF, no HF-MP has been shown to be consistently superior to others [2, 32–35]. It remains unknown what level of investment in health resources is needed for an intervention to be effective. This study will incorporate the latest and most comprehensive RCT evidence to complete the largest review and meta-analysis in the field. In order to reduce clinical heterogeneity, we will conduct an overall classification based on the frequencies of encounters, the primary care provider, and the intensities of content in each study and evaluate the effectiveness of various intervention models. At the same time, combined with the taxonomy provided by the AHA disease management statement [18] and patient data (age, gender, LVEF, etc.), a regression analysis was performed on the intervention details to determine the applicable conditions of different intervention models. Of course, this study may be limited by sample size and methodology and still has high statistical heterogeneity. We may minimize this effect by performing a subgroup analysis and network-meta-regression.

The results of this study will provide the highest level of evidence to assist clinicians, healthcare professionals, and policymakers in developing HF-MPs based on the characteristics of patients with HF.

## Supplementary Information

**Additional file 1: Supplementary Materials.** A. Components of heart failure management plan in this study. B. The specific search strategy for different database.

## Data Availability

Data sharing is not applicable to this article as no datasets were generated or analyzed during the current study.

## Abbreviations

HF: Heart failure
HF-MP: Heart failure management program
ESC: European Society of Cardiology
RCT: Randomized clinical trial
NMA: Network meta-analysis
AHA: American Heart Association
PRISMA-P: Preferred Reporting Items for Systematic Reviews and Meta-analyses Protocols
PICOS: Population-Intervention-Comparison-Outcomes-Study
QOL: Quality of life
KCCQ: Kansas City Cardiomyopathy Questionnaire
MLHF: Minnesota Living with Heart Failure Questionnaire
SF-12: 12-Item Short Form Health Survey
SF-36: 36-Item Short Form Health Survey questionnaire
GRADE: Grading of Recommendations Assessment, Development and Evaluation
High-HDM: High-intensity hybrid disease management
Low-HDM: Low-intensity hybrid disease management
High-SCS: High-intensity self-care support
Low-SCS: Low-intensity self-care support
SM: Self-monitoring
UC: Usual care
EUC: Enhanced usual care
HR: Hazard ratio
OR: Odds ratio
SMD: Standardized mean difference
SUCRA: Surface under the cumulative ranking curve
