# Supplementary material for: Efficacy of disease management program used among patients with chronic heart failure: protocol for a systematic review and network meta-analysis
Source: Syst Rev. 2023 Feb 28;12:27. doi: 10.1186/s13643-023-02183-8 (PMC9972626; doi:10.1186/s13643-023-02183-8)
Supplement: Supplementary file 1 — Additional file 1: Supplementary Materials. A. Components of heart failure management plan in this study. B. The specific search strategy for different database. [file 13643_2023_2183_MOESM1_ESM.docx]

**Supplementary Materials**

**A. Components of heart failure management plan in this study**

**Table A.** **Components of heart failure management plan**

| Components |
| --- |
| 1. Optimized management; lifestyle choices, pharmacological, and devices |
| 2. Patient education, with special emphasis on self-care and symptom management |
| 3. Provision of psychosocial support to patients and family caregivers |
| 4. Follow-up after discharge (clinic; home visits; telephone support or telemonitoring) |
| 5. Easy access to healthcare, especially to prevent and manage decompensation |
| 6. Assessment of (and appropriate intervention in response to) an unexplained change in weight, nutritional and functional status, quality of life, sleep problems, psychosocial problems or other findings (e.g., laboratory values) |
| 7. Access to advanced treatment options; supportive and palliative care |

**B. The specific search strategy for different database**

**1.Ovid MEDLINE(R) and In-Process, In-Data-Review & Other Non-Indexed Citations 2000 to August 31, 2021, Database Field Guide Ovid MEDLINE(R) Epub Ahead of Print August 31, 2021**

- 1. heart failure.ti.
  2. acute heart failure.ti,ab.
  3. acute.ti.
  4. 2 or 3
  5. 1 not 4
  6. randomized controlled trial.pt.
  7. controlled clinical trial.pt.
  8. (random$ or group).ti,ab.
  9. (meta analysis or review or case report).ti.
  10. (6 or 7 or 8) not 9
  11. self administration/
  12. self care/
  13. self medication/
  14. self help groups/
  15. Patient Education/
  16. Telemedicine/
  17. Remote Consultation/
  18. Case Management/
  19. After care/
  20. discharge education/
  21. Continuity of Patient Care/
  22. transitional care/
  23. (after care or aftercare).ti,ab.
  24. telemedicine.ti,ab.
  25. telecare.ti,ab.
  26. telenursing.ti,ab.
  27. telemonitor$.ti,ab.
  28. telehealth.ti,ab.
  29. Action plan$.ti,ab.
  30. (telephon$ or phone$ or remote or nurs$ or tablet or Internet or app).ti,ab.
  31. home$.ti,ab.
  32. (self care or selfcare).ti,ab.
  33. (self manag$ or selfmanag$).ti,ab.
  34. (self monitor$ or selfmonitor$).ti,ab.
  35. (self help or selfhelp).ti,ab.
  36. (self diagnos$ or selfdiagnos$ or self assess$ or selfassess$).ti,ab.
  37. (self treat$ or selftreat$ or self cure or selfcure).ti,ab.
  38. (self medicat$ or selfmedicat$ or self remed$ or selfremed$).ti,ab.
  39. (case management or management program$).ti,ab.
  40. (Management adj2 (plan$ or program)).ti,ab.
  41. ((Management or care) adj2 (plan$ or program)).ti,ab.
  42. patient education.ti,ab.
  43. ((self administer$ adj2 questionnaire$) or (self administer$ adj2 survey$) or (selfadminister$ adj2 interview$)).ti,ab.
  44. ((pharmacist$ or pharmacy or pharmacies) adj2 (support$ or assist$ or advice or advis$ or inform$)).ti,ab.
  45. pharmaceutical care.ti,ab.
  46. (group adj1 (support$ or advice or advis$ or monitor$ or intervention$ or train$ or instruction or consult$ or assist$ or education or educate or information)).ti,ab.
  47. ((telephon$ or remote or phone) adj2 (follow$ or support or consult$ or advice or advis$ or intervention$ or train$ or instruction or assis$ or educate or education or information or monitor$)).ti,ab.
  48. (nurse adj2 educat$).ti,ab.
  49. (patient adj2 (education or advice or advis$ or instruct$ or educate or train$)).ti,ab.
  50. 11 or 12 or 13 or 14 or 15 or 16 or 17 or 18 or 19 or 20 or 21 or 22 or 23 or 24 or 25 or 26 or 27 or 28 or 29 or 30 or 31 or 32 or 33 or 34 or 35 or 36 or 37 or 38 or 39 or 40 or 41 or 42 or 43 or 44 or 45 or 46 or 47 or 48 or 49
  51. 5 and 10 and 50
  52. limit 51 to (english language and yr="2000 -Current")
  53. (animals not (humans and animals)).sh.
  54. 52 not 53

**2.Cochrane Library**

- 1. "heart failure":ti
  2. "acute heart failure":ti,ab
  3. acute:ti
  4. #2 OR #3
  5. #1 NOT #4
  6. "randomized controlled trial":pt
  7. "controlled clinical trial":pt
  8. (random*:ti,ab OR group:ti,ab)
  9. ("meta analysis":ti OR review:ti OR "case report":ti)
  10. (#6 OR #7 OR 8 ) NOT #9
  11. [mh ^"self administration"]
  12. [mh ^"self care"]
  13. [mh ^"self medication"]
  14. [mh ^"self help groups"]
  15. [mh ^"Patient Education"]
  16. [mh ^Telemedicine]
  17. [mh ^"Remote Consultation"]
  18. [mh ^"Case Management"]
  19. [mh ^"After care"]
  20. [mh ^"Continuity of Patient Care"]
  21. [mh ^"transitional care"]
  22. ("after care":ti,ab OR aftercare:ti,ab)
  23. telemedicine:ti,ab
  24. telecare:ti,ab
  25. telenursing:ti,ab
  26. telemonitor*:ti,ab
  27. telehealth:ti,ab
  28. ("Action" NEXT plan*):ti,ab
  29. (telephon*:ti,ab OR phone*:ti,ab OR remote:ti,ab OR nurs*:ti,ab OR tablet:ti,ab OR Internet:ti,ab OR app:ti,ab)
  30. home*:ti,ab
  31. ("self care":ti,ab OR selfcare:ti,ab)
  32. (("self" NEXT manag*):ti,ab OR selfmanag*:ti,ab)
  33. (("self" NEXT monitor*):ti,ab OR selfmonitor*:ti,ab)
  34. ("self help":ti,ab OR selfhelp:ti,ab)
  35. (("self" NEXT diagnos*):ti,ab OR selfdiagnos*:ti,ab OR ("self" NEXT assess*):ti,ab OR selfassess*:ti,ab)
  36. (("self" NEXT treat*):ti,ab OR selftreat*:ti,ab OR "self cure":ti,ab OR selfcure:ti,ab)
  37. (("self" NEXT medicat*):ti,ab OR selfmedicat*:ti,ab OR ("self" NEXT remed*):ti,ab OR selfremed*:ti,ab)
  38. ("case management":ti,ab OR ("management" NEXT program*):ti,ab)
  39. (Management:ti,ab NEAR/2 (plan*:ti,ab OR program:ti,ab))
  40. ((Management:ti,ab OR care:ti,ab) NEAR/2 (plan*:ti,ab OR program:ti,ab))
  41. "patient education":ti,ab
  42. ((("self" NEXT administer*):ti,ab NEAR/2 questionnaire*:ti,ab) OR (("self" NEXT administer*):ti,ab NEAR/2 survey*:ti,ab) OR (selfadminister*:ti,ab NEAR/2 interview*:ti,ab))
  43. ((pharmacist*:ti,ab OR pharmacy:ti,ab OR pharmacies:ti,ab) NEAR/2 (support*:ti,ab OR assist*:ti,ab OR advice:ti,ab OR advis*:ti,ab OR inform*:ti,ab))
  44. "pharmaceutical care":ti,ab
  45. (group:ti,ab NEAR/1 (support*:ti,ab OR advice:ti,ab OR advis*:ti,ab OR monitor*:ti,ab OR intervention*:ti,ab OR train*:ti,ab OR instruction:ti,ab OR consult*:ti,ab OR assist*:ti,ab OR education:ti,ab OR educate:ti,ab OR information:ti,ab))
  46. ((telephon*:ti,ab OR remote:ti,ab OR phone:ti,ab) NEAR/2 (follow*:ti,ab OR support:ti,ab OR consult*:ti,ab OR advice:ti,ab OR advis*:ti,ab OR intervention*:ti,ab OR train*:ti,ab OR instruction:ti,ab OR assis*:ti,ab OR educate:ti,ab OR education:ti,ab OR information:ti,ab OR monitor*:ti,ab))
  47. (nurse:ti,ab NEAR/2 educat*:ti,ab)
  48. (patient:ti,ab NEAR/2 (education:ti,ab OR advice:ti,ab OR advis*:ti,ab OR instruct*:ti,ab OR educate:ti,ab OR train*:ti,ab))
  49. #11 OR #12 OR #13 OR #14 OR #15 OR #16 OR #17 OR #18 OR #19 OR #20 OR #21 OR #22 OR #23 OR #24 OR #25 OR #26 OR #27 OR #28 OR #29 OR #30 OR #31 OR #32 OR #33 OR #34 OR #35 OR #36 OR #37 OR #38 OR #39 OR #40 OR #41 OR #42 OR #43 OR #44 OR #45 OR #46 OR #47 OR #48 OR #49
  50. #5 AND #10 AND #49

**3.PUBMED**

heart failure[Title] AND ((self management[Title/Abstract]) OR (self care[Title/Abstract]) OR (monitor[Title/Abstract]) OR (education[Title]) OR (telemedicine[Title/Abstract]) OR (remote[Title/Abstract]) OR (telephone[Title/Abstract]) OR (telehealth[Title/Abstract])OR (nurse[Title/Abstract]) OR (nursing[Title/Abstract]) OR (monitor*[Title/Abstract]) OR (assist*[Title/Abstract]) OR (telecare[Title/Abstract]) OR (telenurs*[Title/Abstract]) OR (telemonitor*[Title/Abstract]) OR (phone[Title/Abstract]) OR (app[Title/Abstract]) OR (self help[Title/Abstract]) OR (self-guided[Title/Abstract]) OR (self-monitoring[Title/Abstract]) OR (self-administration[Title/Abstract]) OR (self[Title/Abstract]) ) AND ((clinical trial[Title/Abstract]) OR (randomized controlled trials[Title/Abstract]) OR (controlled clinical trial[Title/Abstract]) OR (randomized [Title/Abstract])OR (randomly[Title/Abstract])) NOT ((acute heart failure[Title/Abstract]) OR (acute[Title]))
